# Clinical performance of SARS-CoV-2 antigen-detection rapid diagnostic test using SERS-based lateral flow immunoassay

**DOI:** 10.1016/j.heliyon.2023.e19492

**Published:** 2023-08-29

**Authors:** Sumi Yoon, Yong Kwan Lim, Oh Joo Kweon, Tae-Hyoung Kim, Mi-Kyung Lee

**Affiliations:** a Department of Laboratory Medicine, Chung-Ang University College of Medicine, Seoul, South Korea; b Department of Urology, Chung-Ang University College of Medicine, Seoul, South Korea

**Keywords:** COVID-19, SARS-CoV-2, Antigen-detection rapid diagnostic test, SERS, ACROSIS COVID-19 Ag (NPS), Clinical performance

## Abstract

**Background:**

‘ACROSIS COVID-19 Ag (NPS)’ kit (SG Medical, Seoul, Korea) is a newly developed severe acute respiratory syndrome coronavirus 2 (SARS-CoV-2) antigen-detection rapid diagnostic test (Ag-RDT) using surface-enhanced Raman scattering (SERS)-based lateral flow immunoassay (LFIA). We evaluated its clinical performance compared with STANDARD Q COVID-19 Ag (SD Biosensor, Suwon, Korea), a previously approved Ag-RDT.

**Methods:**

A total of 286 nasopharyngeal swab specimens were collected: 104 positive and 182 negative specimens in SARS-CoV-2 real-time reverse-transcription polymerase-chain-reaction (rRT-PCR). SARS-CoV-2-positive specimens were divided according to the cycle threshold (Ct) value in rRT-PCR. The clinical performance of ACROSIS was compared with that of STANDARD Q.

**Results:**

ACROSIS showed significantly higher sensitivity than STANDARD Q (92.3% vs. 85.6%, *P* = 0.02), especially in specimens with 25 ≤ Ct < 30 (78.6% vs. 42.9%). The Ct values of *RdRp*/*S* genes for 95% detection rates by ACROSIS and STANDARD Q were 25.8 and 23.0, respectively.

**Conclusions:**

This is the first study that evaluated the performance of ACROSIS compared with STANDARD Q. The overall clinical performance of ACROSIS was superior to that of STANDARD Q, especially in specimens with 25 ≤ Ct < 30. ACROSIS could be useful for SARS-CoV-2 Ag detection even in relatively low viral load specimens.

## 1 Introduction

Coronavirus disease 2019 (COVID-19), an infectious disease caused by severe acute respiratory syndrome coronavirus 2 (SARS-CoV-2), has rapidly spread worldwide since it first broke out in Wuhan, China, in December 2019 [1,2]. The World Health Organization (WHO) declared COVID-19 pandemic on March 11, 2020; more than two years later, the pandemic continues and is a significant concern for public health [1,3]. The molecular test such as real-time reverse-transcription polymerase-chain-reaction (rRT-PCR) is still considered the gold standard for detecting SARS-CoV-2 RNA in respiratory tract specimens [4]. Various SARS-CoV-2 rRT-PCR assays are widely used in clinical practice [5]. However, rRT-PCR has several disadvantages. It is expensive, requires sophisticated laboratory infrastructure, and depends on skilled laboratory personnel [[6], [7], [8]]. In addition, the long turnaround time of rRT-PCR could delay a prompt diagnosis of SARS-CoV-2 infection, resulting further transmission [[7], [8], [9]].

In addition to rRT-PCR, the WHO recommends antigen-detection rapid diagnostic tests (Ag-RDTs) as a reliable option for diagnosis of SARS-CoV-2 infection [10,11]. Ag-RDTs are less expensive, faster, and available as point-of-care (POC) tests, which can be useful for SARS-CoV-2 surveillance, especially in low- and middle-income countries [[6], [7], [8], [9]]. Since mid-2020, many SARS-CoV-2 Ag-RDTs, including STANDARD Q COVID-19 Ag (STANDARD Q; SD Biosensor, Suwon, Korea), GenBody COVID-19 Ag (GenBody America, Jurupa Valley, CA, USA), and BIOCREDIT COVID-19 Ag (RapiGEN, Suwon, Korea), have been developed and approved by public health authorities or international regulatory agencies [6,[10], [11], [12]]. The WHO recommends that Ag-RDTs meet performance requirements of ≥80% sensitivity and ≥97% specificity in cases suspected of SARS-CoV-2 infection, and should be used preferentially in symptomatic individuals [10,11]. In Korea, SARS-CoV-2 Ag-RDTs are not recommended for asymptomatic individuals and are considered clinically efficacious if they achieved a sensitivity of ≥80% and a specificity of ≥95% [13,14]. Many studies have evaluated the performance of SARS-CoV-2 Ag-RDTs, with variable results [[6], [7], [8], [9],[15], [16], [17], [18], [19], [20], [21], [22], [23], [24]]. Most Ag-RDTs, including STANDARD Q, are based on the lateral flow immunoassay (LFIA) and are less sensitive than rRT-PCR, especially in low viral load specimens, which may cause some early COVID-19 patients to be missed [8,[15], [16], [17], [18]].

A SARS-CoV-2 Ag-RDT using surface-enhanced Raman scattering (SERS)-based LFIA, ‘ACROSIS COVID-19 Ag (NPS)’ kit (ACROSIS; SG Medical, Seoul, Korea), has been newly developed for use by clinical professionals and approved by the Korean Ministry of Food and Drug Safety (MFDS) on June 8, 2023 [12]. It is designed to improve sensitivity using gold nanoparticle complexes and a blank pad [25,26]. To the best of our knowledge, the performance of a SARS-CoV-2 Ag-RDT using SERS-based LFIA, ACROSIS, has not been evaluated. In this study, we aimed to evaluate the clinical performance of ACROSIS using nasopharyngeal swab (NPS) specimens confirmed positive or negative for SARS-CoV-2 on rRT-PCR. We also compared the clinical performance of ACROSIS with that of STANDARD Q, which is the first SARS-CoV-2 Ag-RDT approved by the Korean MFDS and is widely used [10].

## 2 Materials and methods

### 2.1 Specimens

We randomly selected and collected NPS specimens from subjects who visited the Chung-Ang University Medical Center (CAUMC), Seoul, Korea, for SARS-CoV-2 rRT-PCR testing between August 2021 and May 2022. This study was conducted according to the Declaration of Helsinki. The Institutional Review Board (IRB) of the CAUMC approved this study protocol (IRB No. 2204-012-503). As this study was conducted using residual specimens after the requested SARS-CoV-2 rRT-PCR, informed consent was waived according to the IRB policy. In total, 286 NPS specimens were collected from 286 subjects; 104 specimens were confirmed to be SARS-CoV-2-positive, and 182 specimens to be SARS-CoV-2-negative in rRT-PCR. The SARS-CoV-2-positive specimens were divided into three groups according to the number of days the specimens were collected after symptom onset (DASO) (1–3 days, n = 38; 4–7 days, n = 42; ≥8 days, n = 24) and four groups according to the cycle threshold (Ct) value of RNA-dependent RNA polymerase (*RdRp*)/spike (*S*) genes in rRT-PCR (Ct < 20, n = 44; 20 ≤ Ct < 25, n = 35; 25 ≤ Ct < 30, n = 14; Ct ≥ 30, n = 11). NPS specimens were collected in the Clinical Virus Transport Medium (VTM) (UTNFS-3B-2; Noble Biosciences, Inc., Hwaseong, Korea) and stored at −70 °C until testing. The data were analyzed anonymously.

### 2.2 Ag-RDTs and rRT-PCR for detection of SARS-CoV-2

**Fig. 1:**
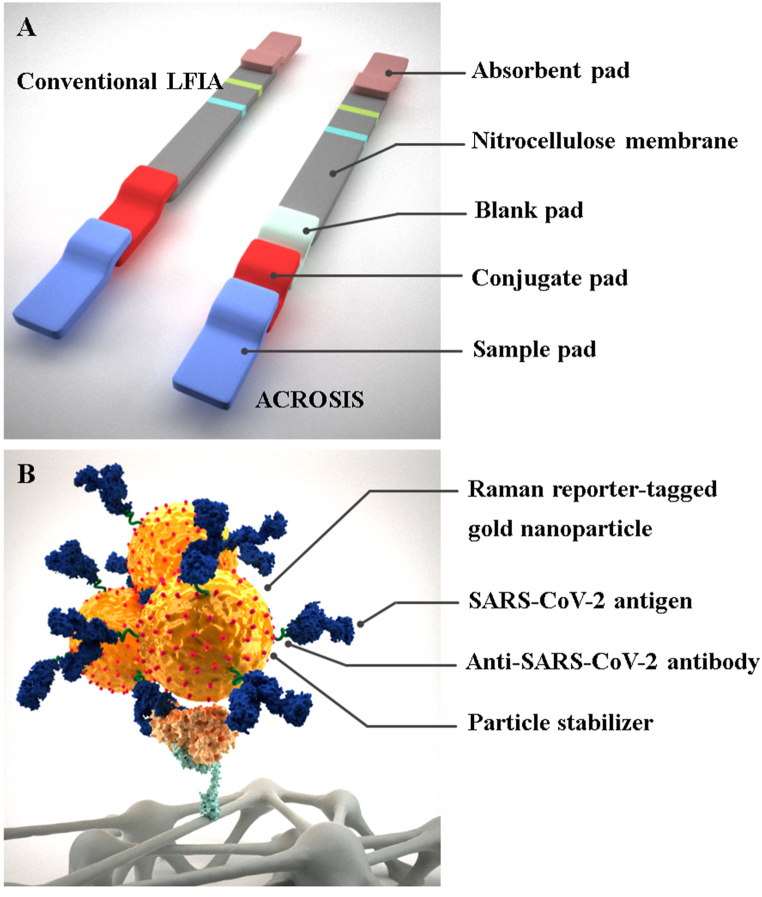
Principle of ACROSIS. (A) The construction of ACROSIS device comparison to conventional lateral flow immunoassay (LFIA). The blank pad that increases the reaction time between *anti*-SARS-CoV-2 antibodies and antigens. (B) The complex structure of gold nanoparticles clustered by Raman reporter.

All specimens were tested for one day in parallel using two Ag-RDTs, ACROSIS and STANDARD Q. The manufacturers of both Ag-RDTs recommend two types of specimens: direct NPS specimens and those stored in VTM. In this study, the NPS specimens stored in VTM (300 μL and 350 μL in ACROSIS and STANDARD Q, respectively) were mixed with the extraction buffers provided with each test kit, according to the manufacturer's instructions [27]. Both Ag-RDTs detect the nucleocapsid protein of SARS-CoV-2 in human NPS specimens based on a lateral flow immunochromatographic assay [20]. The specimen mixed with the extraction buffers flows through the pad and membrane of the device by capillarity. SARS-CoV-2 Ag in the specimen interact with *anti*-SARS-CoV-2 antibody (Ab) conjugated with color particles in the conjugate pad. This complex migrates along the nitrocellulose membrane until it reaches the test line, where it is captured by the *anti*-SARS-CoV-2 Ab (Fig. 1A) [27]. Unlike conventional LFIA, including STANDARD Q, ACROSIS integrates Raman reporter-labeled nanoparticles (SERS nanotags) into the LFIA as detection probes [28]. In addition, it is designed to improve the relatively low sensitivity of the LFIA by using gold nanoparticle complexes and inserting a blank pad [25,26]. The complex structure of gold nanoparticles is formed by malachite green isothiocyanate as a surface bound Raman reporter and may improve the sensitivity due to a large number of nanoparticles per unit area (Fig. 1B) [25]. The blank pad is located between the conjugate pad and the nitrocellulose membrane to increase the reaction time between the *anti*-SARS-CoV-2 Ab conjugated with gold nanoparticles in the conjugate pad and the SARS-CoV-2 Ag (Fig. 1A) [26]. According to the manufacturer's instructions, the limit of detection (LoD) of ACROSIS is determined as 2.37 × 10^5^ 50% tissue culture infectious dose (TCID_50_)/mL for direct NPS specimens and 4.75 × 10^6^ TCID_50_/mL for the NPS specimens stored in VTM. ACROSIS has a kit stability period of 24 months. Regarding sample stability, it ensures sample integrity for a period of 12 months. For comparison with ACROSIS, STANDARD Q was used as a conventional SARS-CoV-2 Ag-RDT in this study. According to the manufacturer's instructions, the LoD of STANDARD Q is determined as 3.12 × 10^2.2^ TCID_50_/mL for direct NPS specimens and 5 × 10^3.2^ TCID_50_/mL for the NPS specimens stored in VTM. STANDARD Q has a stability period of 24 months [27].

For the requested SARS-CoV-2 rRT-PCR, nucleic acids were extracted from NPS specimens using the NucliSens easyMAG System (bioMérieux SA, Marcy l'Etoile, France). The rRT-PCRs were performed using the Allplex™ SARS-CoV-2 Assay (Seegene, Inc., Seoul, Korea) on the CFX96™ Real-Time PCR Detection System (Bio-Rad, Hercules, CA, USA) according to the manufacturer's instructions. The Allplex™ SARS-CoV-2 Assay detects four target genes: the envelope (*E*) gene common to all sarbecoviruses, the nucleocapsid (*N*), *RdRp*, and *S* genes specific to SARS-CoV-2. The rRT-PCR result was interpreted as SARS-CoV-2-positive when the Ct value for all target genes was ≤40 and SARS-CoV-2-negative when the Ct value for all target genes was >40; other results were interpreted as inconclusive.

### 2.3 Statistical analysis

The clinical performance of ACROSIS and STANDARD Q was evaluated using sensitivity, specificity, positive predictive value (PPV), and negative predictive value (NPV) based on rRT-PCR results. The sensitivity and specificity of ACROSIS and STANDARD Q were compared using McNemar's Chi-squared test for paired proportions. For SARS-CoV-2-positive specimens, the sensitivity was evaluated according to the number of DASO and the Ct value for *RdRp*/*S* genes. Since there was no significant difference in Ct values for each gene in the rRT-PCR (all *P* > 0.05), the Ct value of any gene could be used for analysis. We selected *RdRp/S* genes that are specific to SARS-CoV-2 and are detected in the same fluorescence channel [29]. In addition, the clinical performance was evaluated using the binomial logistic regression analysis [30]. The logistic regression model was constructed based on the binomial results of each Ag-RDT and the Ct value for *RdRp*/*S* genes in the rRT-PCT test. The relationship between the Ag-RDT results and the Ct value could be predicted using this analysis. The Ct values (Ct_50_ and Ct_95_) for *RdRp*/*S* genes were estimated when 50% and 95% detection rates were achieved in each Ag-RDT. The correlation between ACROSIS and STANDARD Q was evaluated using positive percent agreement, negative percent agreement, and overall percent agreement (OPA). Cohen's kappa (κ) with 95% confidence interval (CI) was calculated and interpreted as follows: ≤0.20, none; 0.21–0.39, minimal; 0.40–0.59, weak; 0.60–0.79, moderate; 0.80–0.90, strong; >0.90, nearly perfect [31]. Statistical analyses were performed using MedCalc Statistical Software (version 20.109; MedCalc Software, Ostend, Belgium). *P* < 0.05 was considered statistically significant.

## 3 Results

**Fig. 2:**
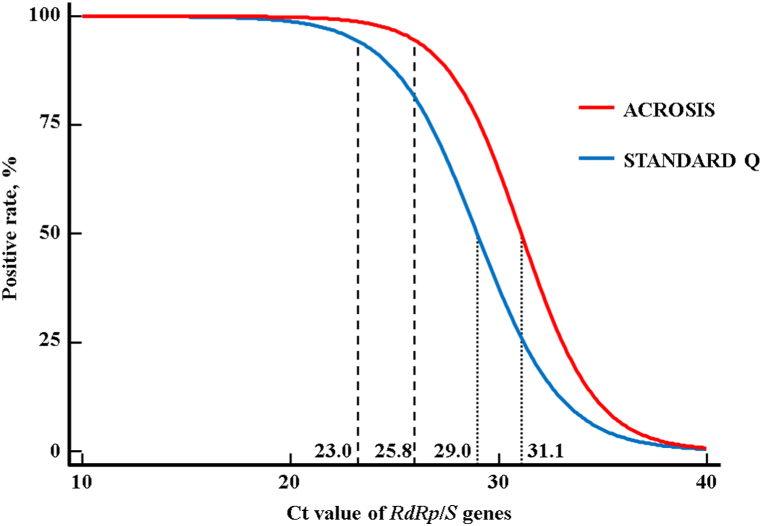
Logistic regression analyses for the positive rate of ACROSIS and STANDARD Q according to the Ct value for *RdRp*/*S* genes in Allplex™ SARS-CoV-2 Assay. The dotted and dashed lines represent the Ct values (Ct_50_ and Ct_95_) when 50% and 95% detection rates are achieved in each Ag-RDT.

The overall sensitivities of ACROSIS and STANDARD Q were 92.3% (96/104) and 85.6% (89/104), respectively; the overall sensitivity of ACROSIS was statistically significantly higher than that of STANDARD Q (*P* = 0.02). The overall specificity of both Ag-RDTs was 100.0% (95% CI, 98.0%–100.0%); thus, McNemar's Chi-squared test to compare their specificity could not be performed. The PPV of both Ag-RDTs was 100.0%, and the NPVs of ACROSIS and STANDARD Q were 95.8% (95% CI, 92.1%–97.8%) and 92.4% (95% CI, 88.4%–95.1%), respectively. In the group divided by the number of DASO, the sensitivity of ACROSIS ranged from 90.5% to 94.7%. The sensitivity of STANDARD Q ranged from 83.3% to 86.8%. In the group divided by the Ct value for *RdRp*/*S* genes, the sensitivity of ACROSIS ranged from 54.5% to 100.0%. The sensitivity of STANDARD Q ranged from 42.9% to 100.0%. Especially, the sensitivity of ACROSIS was significantly higher than that of STANDARD Q in the specimens with 25 ≤ Ct < 30 (78.6% vs. 42.9%, respectively). In each Ag-RDT, there was no significant difference in sensitivity according to the number of DASO, whereas the sensitivity tended to decrease as the Ct value increased (Table 1). The binomial logistic regression analysis showed that the Ct_50_ values of ACROSIS and STANDARD Q for the *RdRp*/*S* genes were 31.1 and 29.0, respectively; the Ct_95_ values were 25.8 and 23.0, respectively (Fig. 2). ACROSIS and STANDARD Q showed a high OPA of 97.6% (95% CI, 95.0–99.0) and a strong agreement (κ = 0.944; 95% CI, 0.903–0.985) (Table 2).

**Table 1:** Clinical sensitivity of ACROSIS and STANDARD Q.

	SARS-CoV-2 (+)^c^			Sensitivity, % (95% CI)
	Total, n (%)	Ag (+), n (%)	Ag (−), n (%)	
**ACROSIS**				
Overall	104 (100.0)	96 (92.3)	8 (7.7)	92.3 (85.4–96.6)
Number of days^a^				
1–3	38 (36.5)	36 (34.6)	2 (1.9)	94.7 (82.3–99.4)
4–7	42 (40.4)	38 (36.5)	4 (3.8)	90.5 (77.4–97.3)
≥8	24 (23.1)	22 (21.2)	2 (1.9)	91.7 (73.0–99.0)
Ct value^b^				
Ct < 20	44 (42.3)	44 (42.3)	0 (0.0)	100.0 (92.0–100.0)
20 ≤ Ct < 25	35 (33.7)	35 (33.7)	0 (0.0)	100.0 (90.0–100.0)
25 ≤ Ct < 30	14 (13.5)	11 (10.6)	3 (2.9)	78.6 (49.2–95.3)
Ct ≥ 30	11 (10.6)	6 (5.8)	5 (4.8)	54.5 (23.4–83.3)
**STANDARD Q**				
Overall	104 (100.0)	89 (85.6)	15 (14.4)	85.6 (77.3–91.7)
Number of days^a^				
1–3	38 (36.5)	33 (31.7)	5 (4.8)	86.8 (71.9–95.6)
4–7	42 (40.4)	36 (34.6)	6 (5.8)	85.7 (71.5–94.6)
≥8	24 (23.1)	20 (19.2)	4 (3.8)	83.3 (62.6–95.3)
Ct value^b^				
Ct < 20	44 (42.3)	44 (42.3)	0 (0.0)	100.0 (92.0–100.0)
20 ≤ Ct < 25	35 (33.7)	34 (32.7)	1 (1.0)	97.1 (85.1–99.9)
25 ≤ Ct < 30	14 (13.5)	6 (5.8)	8 (7.7)	42.9 (17.7–71.1)
Ct ≥ 30	11 (10.6)	5 (4.8)	6 (5.8)	45.5 (16.7–76.6)

Abbreviations: Ag, antigen; CI, confidence interval; Ct, cycle threshold; n, number; *RdRp*, RNA-dependent RNA polymerase; *S*, spike; SARS-CoV-2, severe acute respiratory syndrome coronavirus 2.

a The number of days the specimens were collected after symptom onset.

b Ct value for the *RdRp*/*S* genes in Allplex™ SARS-CoV-2 Assay.

c SARS-CoV-2-positive and -negative were confirmed by Allplex™ SARS-CoV-2 Assay.

**Table 2:** Agreement between ACROSIS and STANDARD Q (n = 286).

ACROSIS	STANDARD Q		Positive percent agreement, % (95% CI)	Negative percent agreement, % (95% CI)	Overall percent agreement, % (95% CI)	Kappa (95% CI)
	Positive, n	Negative, n				
Positive	89	7	100.0 (95.9–100.0)	96.4 (92.8–98.6)	97.6 (95.0–99.0)	0.944 (0.903–0.985)
Negative	0	190				

Abbreviations: CI, confidence interval; n, number.

## 4 Discussion

Although Ag-RDTs are currently widely used to diagnose SARS-CoV-2 infection, there are still concerns that they are relatively low in sensitivity and may provide false negative results [12,23]. SERS is a powerful technique that can analyze single-molecule level sensitivity and quantitative detection capacity; it is applied to numerous fields, including medicine [28]. Many SERS-based platforms have been developed to detect viral or bacterial pathogens, biomarkers, and antibiotics [28,32]. For SARS-CoV-2, a few studies have detected SARS-CoV-2 Ag and/or Ab with high sensitivity by applying SERS [28,33,34].

In this study, we evaluated the clinical performance of a SARS-CoV-2 Ag-RDT using SERS-based LFIA, ACROSIS, compared with that of STANDARD Q using NPS specimens. Our data demonstrated that the sensitivity of ACROSIS was 100.0% in specimens with Ct < 25; it decreased to 54.5% in specimens with Ct ≥ 30. Above all, the sensitivity of ACROSIS in specimens with 25 ≤ Ct < 30 was 78.6%, significantly higher than that in previous studies [23,[35], [36], [37]]. It has been demonstrated that the application of SERS improves the sensitivity and lowers the detection limit of analytes [28,32]. The detection limits of SERS-based platforms were up to 2000 times more sensitive [32]. Although ACROSIS had a smaller input volume (300 μL) compared to STANDARD Q (350 μL), it demonstrated higher sensitivity than STANDARD Q [27]. Our findings suggest that the SERS-based Ag-RDT could be a useful analytical tool with improved sensitivity even in low viral load specimens. ACROSIS was more sensitive than STANDARD Q in all specimens, in the groups divided by the number of DASO, and in the groups divided by the Ct value for *RdRp*/*S* genes. ACROSIS showed almost half the false negative rate compared with STANDARD Q (7.7% vs. 14.4%, respectively). In the SARS-CoV-2-positive specimens, seven were detected by ACROSIS but could not be detected by STANDARD Q; the Ct values for the *RdRp*/*S* genes were between 24.3 and 30.9. The Ct_50_ and Ct_95_ values of ACROSIS for the *RdRp*/*S* genes determined by the binomial logistic regression analysis were 31.1 and 25.8, respectively, which were higher than those in previous studies [23,36]. Our findings indicate that ACROSIS can detect the specimens with a lower viral load than STANDARD Q. However, contrary to our findings, the LoD of STANDARD Q is lower than that of ACROSIS according to the manufacturer's instructions. It might be related to the different strains of SARS-CoV-2 tested by each manufacturer to evaluate the LoD (ACROSIS, Italy-INMI1; STANDARD Q, NCCP 43326/2020/Korea) [27]. In addition, since the type of rRT-PCR assay used in this study was different from that of in previous studies, further research using different rRT-PCR assays is needed to support our findings.

The strength of this study is that it provides fundamental data on the performance of ACROSIS for clinical application and further research. However, there are several limitations to this study. First, although we included SARS-CoV-2-positive specimens, the number of specimens in each group divided according to the number of DASO or the Ct value for *RdRp*/*S* genes was relatively small. Several studies have reported that Ag-RDTs show significantly higher sensitivity for specimens collected early after symptom onset [20,[38], [39], [40]]. This finding was consistent with the pathophysiology of SARS-CoV-2 that the viral Ag level was high within a week after symptom onset [41]. However, our study showed no significant difference in sensitivity according to the number of DASO. It is necessary to further evaluate ACROSIS with a larger number of specimens consisting of various DASO. Second, we did not include asymptomatic patients who were SARS-CoV-2-positive in rRT-PCR. Ag-RDTs are recommended to test asymptomatic patients at high risk of infection in settings where rRT-PCR testing capacity is limited [10]. The European Center for Disease Prevention and Control recommends Ag-RDTs for screening asymptomatic patients in settings with an estimated prevalence of ≥10% [42]. Therefore, it is important to evaluate the clinical performance of Ag-RDTs for asymptomatic patients. A meta-analysis study reported that the sensitivity of Ag-RDTs was lower in asymptomatic patients [40]. On the other hand, another study reported high sensitivity of 100% in asymptomatic patients, but the number of asymptomatic patients was only four [20]. Further studies are needed to evaluate whether ACROSIS shows high sensitivity even in asymptomatic patients. Third, this study was conducted using residual specimens stored in VTM as per the manufacturer's recommendation. Previous studies have reported that the sensitivity of Ag-RDTs varied from 11.1% to 98.0% according to the Ag-RDT kit, specimen type, and viral load in the specimen [[6], [7], [8],[15], [16], [17], [18], [19], [20],^23^]. The sensitivity was high in NPS specimens with a high viral load, whereas it was significantly low in sputum specimens [15]. Direct NPS specimens collected fresh and mixed with the extraction buffers without dilution in VTM are a requirement for many POC tests [43]. It should be considered that dilution of specimens by VTM may affect the sensitivity of Ag-RDTs. Nevertheless, ACROSIS showed high overall sensitivity and specificity (92.3% and 100.0%, respectively) that satisfied the recommendations of the WHO and the Korean MFDS [10,11,13,14]. In addition, the sensitivity of ACROSIS was higher than that of STANDARD Q in NPS specimens stored equally in VTM. Last, we did not identify the variant type of virus in the SARS-CoV-2-positive specimens. The variants of the virus is one of the crucial factors contributing to the false negative results of SARS-CoV-2 detection [44]. Given that the specimens were collected between August 2021 and May 2022, it could be inferred that the SARS-CoV-2 variants detected in this study were likely the Delta or Omicron (BA.1 and BA.2) variants, which were dominant during that period in Korea [45]. It is necessary to further compare the performance of ACROSIS according to the SARS-CoV-2 variants.

## 5 Conclusion

This is the first study worldwide to evaluate the clinical performance of ACROSIS compared with that of STANDARD Q. The overall clinical performance of ACROSIS was superior to that of STANDARD Q, especially in specimens with 25 ≤ Ct < 30. ACROSIS, the newly developed SARS-CoV-2 Ag-RDT using SERS-based LFIA, could be useful for SARS-CoV-2 Ag detection even in relatively low viral load specimens. The Ag-RDT using SERS-based LFIA could help diagnose SARS-CoV-2 infections that may be missed in the early stages of infection.

## Declarations

### Author contribution statement

Sumi Yoon: Performed the experiments; Analyzed and interpreted the data; Contributed reagents, materials, analysis tools or data; Wrote the paper.

Yong Kwan Lim, Oh Joo Kweon, Tae-Hyoung Kim: Analyzed and interpreted the data; Contributed reagents, materials, analysis tools or data.

Mi-Kyung Lee: Conceived and designed the experiments; Performed the experiments; Contributed reagents, materials, analysis tools or data; Wrote the paper.

### Funding statement

This study was supported by a 10.13039/501100004663National Research Foundation of Korea grant funded by the Korean government (grant No. 2020R1A5A1018052).

### Data availability statement

Data included in article/supp. material/referenced in article.

## Declaration of competing interest

The authors declare that they have no known competing financial interests or personal relationships that could have appeared to influence the work reported in this paper.
